# Efficacy and safety of adjunctive acupuncture for depression and motor symptoms in Parkinson’s disease: study protocol for a randomized controlled trial

**DOI:** 10.3389/fpsyt.2026.1760698

**Published:** 2026-02-16

**Authors:** Kaihao Liao, Jing-Qi Fan, Liangman Xiao, Danxia Gu, Mingda Han, Jingyu Nian, Shunan Wu, Li-Xing Zhuang

**Affiliations:** 1 First Clinical Medical College of Guangzhou University of Chinese Medicine, Guangzhou, China; 2 The First Affiliated Hospital of Guangzhou University of Chinese Medicine, Guangzhou, China

**Keywords:** acupuncture, motor symptoms, Parkinson’s disease, Parkinson’s disease with depression, randomized controlled trial

## Abstract

**Background:**

Depression is one of the most prevalent and disabling non-motor symptoms in Parkinson’s disease (PD), forming a bidirectional relationship with motor dysfunction that worsens quality of life. Pharmacological treatments exhibit limited and inconsistent efficacy, and may lead to adverse interactions. Acupuncture may improve both depressive and motor symptoms by regulating the neuro–immune–endocrine network, but high-quality evidence remains insufficient.

**Objective:**

This study aims to evaluate the efficacy and safety of acupuncture as an adjunctive therapy for depression in PD and to explore potential biological correlates of clinical changes using predefined serum biomarkers.

**Methods:**

In this single-center, evaluator-blinded, randomized controlled trial, 88 patients with PD and comorbid depression will be randomly assigned to an acupuncture group or a waitlist control group. The primary outcome is the change in the Montgomery–Asberg Depression Rating Scale (MADRS) score. Secondary outcomes include motor function, anxiety, sleep quality, and overall quality of life. Exploratory analyses will assess serum inflammatory cytokines, brain-derived neurotrophic factor (BDNF), and kynurenine/tryptophan (KYN/TRP) ratio.

**Expected results:**

We hypothesize that adjunctive acupuncture may improve depressive and motor symptoms compared with the control. Exploratory analyses will examine whether clinical changes are associated with changes in relevant biomarkers.

**Conclusion:**

This study will provide rigorous evidence for acupuncture as an adjunctive therapy, offering a non-pharmacological strategy to optimize the comprehensive management of PD and disrupt the bidirectional emotion–motor interplay.

**Clinical trial registration:**

https://www.chictr.org.cn/, identifier ChiCTR2500113443.

## 1 Introduction

Depression is among the most challenging comorbidities in the management of Parkinson’s disease (PD) (1). As the most common non-motor symptom, it imposes a considerable epidemiological burden. Approximately 38% of patients with PD develop depression (Parkinson’s disease with depression, PD-D) (2), with a prevalence nearly four times that of the general population and up to ten times higher among individuals over 50 years of age.

Notably, depressive symptoms may precede motor manifestations and establish a maladaptive bidirectional relationship with motor impairment (3). Depression exacerbates bradykinesia, rigidity, and postural instability (4), while motor decline is often linked to the onset or worsening of depressive symptoms (5). This mutually reinforcing pathological process independently predicts disability progression, accelerates functional decline and deterioration in quality of life, and substantially increases the risk of dementia, mortality, and suicide (6, 7).

Therefore, depression in PD is not merely a frequent emotional comorbidity but a pivotal driver of overall disease progression and adverse outcomes. It also places an additional burden on families and healthcare systems (8).

However, current pharmacological and psychological interventions face significant limitations in clinical practice. Although selective serotonin reuptake inhibitors (SSRIs) are considered first-line therapy, evidence for their efficacy in PD remains inconclusive (9). Most trials have been limited by small sample sizes and inconsistent findings, and treatment responses show considerable interindividual variability (10).

Long-term polypharmacy further complicates management. For example, combining SSRIs with monoamine oxidase B inhibitors may trigger serotonin syndrome, while medications such as levodopa can induce or worsen non-motor symptoms, including anxiety and insomnia (11). More importantly, most pharmacological agents target only a single symptom domain, limiting their ability to simultaneously improve both motor and affective symptoms (12). Consequently, patient adherence is often poor, and treatment satisfaction remains low.

Psychological interventions, including cognitive behavioral therapy, are recommended in current guidelines for the management of depression in Parkinson’s disease and have demonstrated efficacy in selected patient populations (13). In routine clinical practice, however, their implementation and uptake may be influenced by contextual factors such as therapist availability, cognitive status, treatment intensity, and patient preference. In particular, some patients with PD-related depression present with prominent somatic symptoms or express a preference for interventions targeting physiological regulation, which may affect engagement with purely psychotherapeutic approaches. In this context, there remains a need to explore additional non-pharmacological options that can be used alongside standard care.

The pathogenesis of depression in PD involves multiple pathological pathways shared by both affective and motor symptoms. In addition to dopaminergic dysfunction, impairments in the serotonin and norepinephrine systems play critical roles in emotional regulation and motor control (3). Chronic neuroinflammation, hyperactivation of the hypothalamic-pituitary-adrenal (HPA) axis, and reduced levels of brain-derived neurotrophic factor (BDNF) are closely linked not only to the onset of depression but also to the acceleration of neurodegeneration (9).

Furthermore, disturbances in the kynurenine/tryptophan (KYN/TRP) metabolic pathway decrease the availability of serotonin precursors, facilitating depressive symptoms (14). At the same time, this imbalance promotes the accumulation of neurotoxic metabolites, such as quinolinic acid, which exacerbates dopaminergic neuronal injury and worsens motor dysfunction (15). Together, these findings indicate that depression and motor impairment in PD share convergent pathological mechanisms mediated through neuro–immune–endocrine networks, providing a solid theoretical foundation for integrated therapeutic approaches.

Acupuncture, a safe and widely accepted non-pharmacological therapy, has shown promising potential in the comprehensive management of depression in PD. Evidence from previous studies suggests that acupuncture may influence multiple neurobiological processes relevant to PD-related depression, including neurotransmitter regulation, inflammatory modulation, neurotrophic support, and tryptophan metabolism (16). These mechanisms closely align with the core pathological pathways of PD-related depression, indicating that acupuncture may provide concurrent benefits for both depressive and motor symptoms (17).

However, most existing evidence comes from exploratory studies with small sample sizes, single-center designs, and considerable methodological heterogeneity. As a result, high-quality data that systematically integrate clinical outcomes with objective biological markers remain scarce (18).

Despite these encouraging findings, several key uncertainties remain. Prior studies are often limited by exploratory designs, restricting the precision and interpretability of effect estimates. In addition, most research has relied predominantly on symptom-based outcomes, with limited integration of objective biological markers, leaving it unclear whether observed clinical improvements reflect modulation of underlying neurobiological pathways. Finally, although depression and motor dysfunction in Parkinson’s disease interact bidirectionally, it remains uncertain whether acupuncture is associated with concurrent improvements across both affective and motor domains within the same clinical framework.

Accordingly, we designed a prospective, evaluator-blinded randomized controlled trial to evaluate the efficacy and safety of acupuncture as an adjunct to stable standard pharmacotherapy in patients with Parkinson’s disease and comorbid depression. This study integrates serum biomarkers—including inflammatory cytokines, brain-derived neurotrophic factor (BDNF), and the kynurenine/tryptophan (KYN/TRP) ratio—with clinical outcome measures, enabling an exploratory assessment of whether acupuncture is associated with coordinated improvements in depressive and motor symptoms through shared neuro–immune–endocrine pathways.

## 2 Methods and analysis

### 2.1 Study design

This study will be a single-center, prospective, evaluator- and statistician-blinded, two-arm, parallel-group randomized controlled trial (RCT) that will be conducted at the First Affiliated Hospital of Guangzhou University of Chinese Medicine. We will recruit 88 patients with PD-D, who will be randomly assigned in a 1:1 ratio to either the acupuncture treatment group or the waitlist control group.

All participants will maintain their stable, standard medication regimen throughout the study. A waitlist control design will be used to assess the add-on benefit of acupuncture. For ethical reasons, participants in the control group will be offered compensatory acupuncture treatment after the 6-week control period.

**Figure 1:**
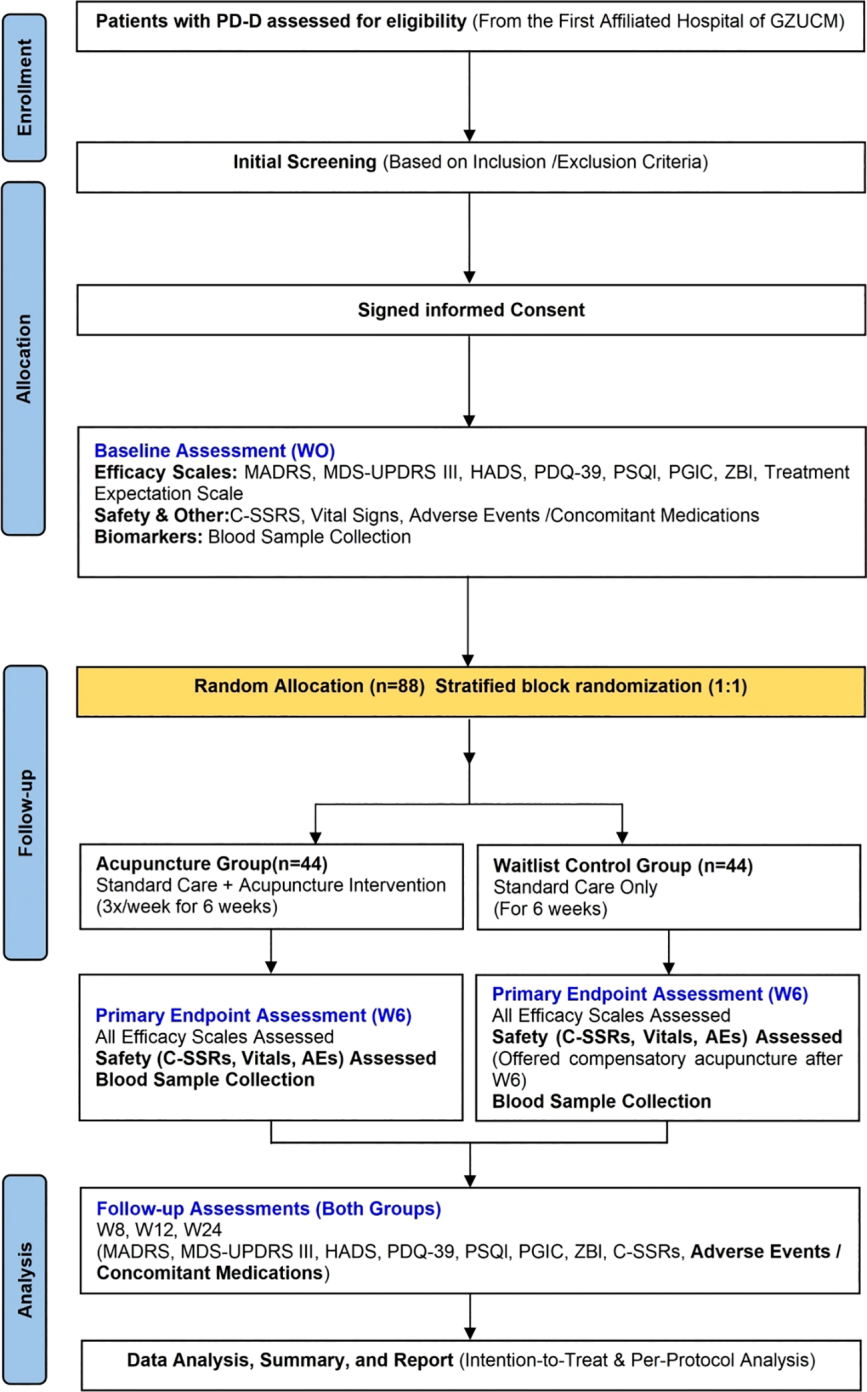
Study flowchart.

The study flow is illustrated in **Figure 1**, outlining the entire process from screening to follow-up. The schedule for all interventions, assessments, and follow-up visits is summarized in **Table 1**. Patients and the public were not involved in the design or conduct of this trial; however, results will be disseminated to participants in plain language after study completion.

**Table 1:** Schedule of enrollment, interventions, and assessments.

Study phase	Assessment content	Screening (W-1 ~ W0)	Baseline (W0)	Primary endpoint (W6)	Follow-up (W8)	Follow-up (W12)	Follow-up (W24)
Enrolment	Eligibility Screening & Informed Consent	✓					
	Demographic & Medical History Information	✓					
	Randomization		✓				
Assessment	MADRS	✓	✓	✓	✓	✓	✓
	HADS		✓	✓	✓	✓	✓
	MDS-UPDRS-III		✓	✓	✓	✓	✓
	PDQ-39		✓	✓	✓	✓	✓
	PSQI		✓	✓	✓	✓	✓
	PGIC			✓	✓	✓	✓
	ZBI		✓	✓	✓	✓	✓
	Acupuncture Expectancy Scale		✓				
	Blood Samples		✓	✓			
Safety	C-SSRS	✓	✓	✓	✓	✓	✓
	Vital Signs¹		✓	✓			
	Adverse Events/Concomitant Medications	✓	✓	✓	✓	✓	✓

¹Vital signs will be assessed at baseline (W0) and at the primary endpoint (W6).

### 2.2 Participants

We will consecutively recruit participants from July 2025 to July 2026 from the acupuncture outpatient clinics and inpatient wards of the First Affiliated Hospital of Guangzhou University of Chinese Medicine. All candidates must be evaluated by a specialist physician, meet the diagnostic criteria for PD-D, and have been on a stable anti-Parkinson’s medication regimen (unchanged dosage and type) for at least 4 weeks before enrollment.

Recruitment will be conducted through multiple channels, including referrals from the outpatient and inpatient departments of Acupuncture, Neurology, and Rehabilitation. On-site promotional materials (e.g., posters, electronic displays) and announcements on the hospital’s official website and WeChat account will also be used.

During the screening phase, trained researchers will systematically collect demographic data (gender, age, education level), medical history (age of onset, disease duration, past and current treatments), and document motor and non-motor symptom characteristics. A trained researcher will explain the study protocol and potential risks to each candidate in a quiet, private setting. Written informed consent will be obtained from all participants after ensuring they fully understand the study and voluntarily agree to participate.

### 2.3 Diagnostic criteria

Currently, there are no internationally accepted diagnostic criteria specifically for depression in PD-D. Therefore, the diagnosis in this study will be based on the following established standards:

Diagnosis of PD: Participants must meet the 2015 clinical diagnostic criteria set by the Movement Disorders Society (MDS) (19).

Diagnosis of Depressive Disorder: Participants must meet the criteria for depressive disorders as outlined in the Diagnostic and Statistical Manual of Mental Disorders, Fifth Edition (DSM-5), published by the American Psychiatric Association.

All diagnoses will be independently made by a qualified neurologist or psychiatrist.

#### 2.3.1 Inclusion criteria

Participants meeting the following criteria will be included:

1. Meet the 2015 MDS clinical diagnostic criteria for PD and the DSM-5 diagnostic criteria for depressive disorder (19).
2. Aged 40–80 years, inclusive, of either gender.
3. Hoehn–Yahr stage 1–3 (20).
4. A baseline MADRS total score between 7 and 34, inclusive, indicating mild to moderate depressive symptoms.
5. Have received antiparkinsonian medication for ≥6 months and have been on a stable dosage for ≥4 weeks prior to enrollment.
6. Stable vital signs, no impaired consciousness, and the ability to communicate normally and complete all study assessments.
7. Voluntarily agree to participate and provide written informed consent.

#### 2.3.2 Exclusion criteria

Participants will be excluded if they meet any of the following criteria:

1. Concurrent severe psychiatric or neurological disorders (e.g., schizophrenia, bipolar disorder, severe cerebrovascular disease).
2. Initiation, discontinuation, or adjustment of antidepressant medications within 4 weeks prior to enrollment; or receipt of any specific intervention for depression (e.g., acupuncture, electroacupuncture, rTMS, psychotherapy) within 4 weeks prior to screening.
3. Concurrent severe systemic diseases (e.g., severe cardiovascular disease, hepatic or renal failure, malignancy), or any condition that may significantly interfere with inflammatory marker assessment (e.g., active infection, regular use of NSAIDs).
4. Significant suicide risk or a recent history of suicidal behavior, as determined by the Columbia-Suicide Severity Rating Scale (C-SSRS).
5. Pregnant or lactating women, or individuals planning pregnancy during the trial period.
6. Known contraindications to acupuncture (e.g., severe needle phobia, history of acupuncture-related syncope, bleeding tendencies, localized infection, or severe arrhythmia).
7. Presence of an implanted deep-brain stimulation (DBS) device.
8. History of alcohol or substance abuse within the last 3 months.

#### 2.3.3 Dropout and discontinuation criteria

Participants will be withdrawn from the study if any of the following criteria are met. The reason for withdrawal will be documented in detail and included in the final analysis. Withdrawal will not affect access to routine clinical care.

Occurrence of a serious adverse event (SAE), or significant worsening of psychiatric symptoms, defined as either (1) an increase in the MADRS total score of more than 50% compared with baseline, or (2) the emergence of clinically significant suicide risk as assessed by the Columbia–Suicide Severity Rating Scale (C-SSRS), or the development of a serious concomitant illness, if the investigator deems continuation inappropriate.

Clinically significant motor worsening is additionally defined as safety-driven deterioration requiring urgent escalation of antiparkinsonian therapy (e.g., new initiation or a clinically necessary substantial dose increase), hospitalization for motor complications, or recurrent injurious falls requiring medical attention; when feasible, a clinically meaningful increase in the MDS-UPDRS III score will be recorded to support documentation.

Inability to continue due to poor adherence, frequent missed visits, or loss to follow-up (e.g., failure to complete ≥2 consecutive follow-up visits).

Any other circumstance deemed unsuitable for continued participation based on a comprehensive assessment by the study team.

### 2.4 Sample size

The sample size was estimated using an Analysis of Covariance (ANCOVA) for the primary outcome: the change in MADRS score from baseline (W0) to Week 6 (W6). Based on prior exploratory research (21) and the conversion formula by Leucht et al. (22), the pre-specified between-group difference (Δ) was set at approximately 2.4 points. An assumed common standard deviation (SD) of approximately 6.0 points for MADRS change scores was adopted, which is consistent with reported variability in previous studies of depression in Parkinson’s disease. This corresponds to a standardized effect size (Cohen’s d) of approximately 0.40. Statistical parameters were set at α = 0.05 (two-tailed) and a power of 1-β = 80%. The calculation, performed using G*Power 3.1 (23), indicated that 35 completers per group are required. To account for a potential 20% dropout rate, we plan to recruit 44 participants per group, resulting in a total sample size of 88. Although the primary analysis will use a mixed-effects model for repeated measures (MMRM), the sample size was estimated using an ANCOVA framework as a conservative approximation for the baseline-adjusted between-group comparison at Week 6.

### 2.5 Randomization and allocation concealment

This study will use a centralized randomization system to ensure unpredictability and allocation concealment. Eligible participants will be randomized in a 1:1 ratio to either the acupuncture group or the waitlist control group. Stratified block randomization will be employed to ensure baseline balance for key prognostic factors. The stratification factors are: (1) baseline depression severity (MADRS ≤ 19 vs. >19) and (2) stable antidepressant medication use (Yes vs. No). The randomization sequence will be automatically generated by the central system. After a participant provides informed consent and completes the baseline assessment, the investigator will retrieve the group assignment from the system and record it on the case report form (CRF).

### 2.6 Blinding

Given the nature of the acupuncture intervention, it is not feasible to blind participants or acupuncturists. Therefore, to minimize bias, this study will employ a single-blind (assessor-blind) design, implementing the following measures:

1. Blinding of Key Personnel: All outcome assessors, data managers, and statisticians will remain blinded to treatment assignment until the database is locked.
2. Separation of Roles: Acupuncturists will not participate in outcome assessment or data management. Conversely, outcome assessors will not be involved in the intervention process to prevent unintentional bias. Acupuncturists will receive standardized training before the trial and adhere to a communication protocol with participants to avoid disclosing any group-related information.
3. Emergency Unblinding Procedure: In the event of a serious adverse event (SAE) or other urgent medical situations where knowledge of group assignment is necessary for participant management, the principal investigator must request approval from the ethics committee. Upon approval, an independent, unblinded data administrator will perform the unblinding. The reason, time, and personnel involved in the unblinding will be fully documented. The unblinded participant will discontinue the study intervention but will continue safety follow-up. Data from unblinded participants will remain in the primary analysis according to the intention-to-treat principle.
4. To further minimize expectancy-related bias, participants’ baseline expectations regarding acupuncture will be assessed using the Acupuncture Expectancy Scale. Expectancy scores will be included as covariates in the primary statistical analysis.
5. To formally evaluate the success of assessor blinding, after completion of the primary endpoint assessment at Week 6 (and, if applicable, at Week 24), blinded outcome assessors will be asked to guess each participant’s treatment allocation (acupuncture or waitlist control) and to indicate their confidence level. Blinding success will be evaluated descriptively by calculating (1) the proportion of correct guesses relative to chance level and (2) Bang’s Blinding Index (BBI) with corresponding 95% confidence intervals. A BBI value close to zero will be interpreted as indicating successful blinding. These analyses are intended to assess the adequacy of blinding procedures rather than to test treatment effects.

### 2.7 Interventions

#### 2.7.1 Acupuncture group

In addition to their standard pharmacotherapy, participants assigned to the acupuncture group will receive a standardized acupuncture intervention. The treatment protocol consists of 18 sessions, administered three times per week over 6 weeks.

All procedures will be performed by licensed physicians with at least 3 years of clinical acupuncture experience. Prior to the trial, all acupuncturists must complete standardized training and pass an assessment to ensure procedural consistency. To ensure continuity, each participant will be treated by the same acupuncturist throughout the intervention period.

**Figure 2:**
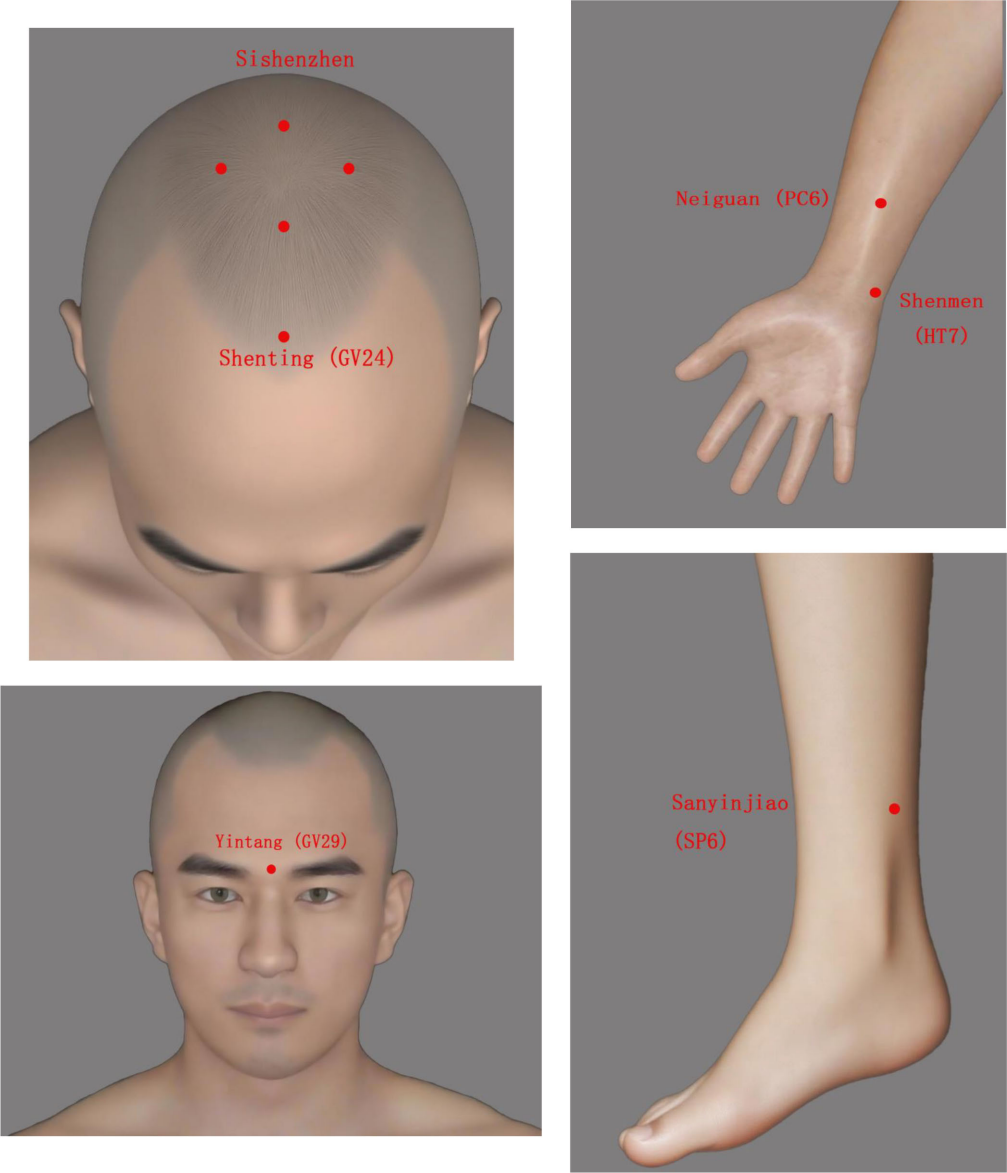
The location of each acupoint.

Acupoint Prescription and Needles:

The selected acupoints are: Sishenzhen(four points located 1.5 cun anterior, posterior, and lateral to GV20), Shenting (GV24), Yintang (GV29), Neiguan (PC6, bilateral), Shenmen (HT7, bilateral), and Sanyinjiao (SP6, bilateral). Please refer to **Figure 2** for the location of the related acupoints.

The acupoint prescription was informed by traditional acupuncture principles and supported by neuroanatomical plausibility. Sishenzhen, Shenting (GV24), and Yintang (GV29) were selected as core points to “regulate Spirit (Shen)” along the Governor Vessel. Anatomically, these scalp points overlie cortical projection areas related to the prefrontal cortex, a key region involved in affect regulation, as suggested by functional neuroimaging studies examining acupuncture-related modulation of prefrontal and affective brain networks (24, 25). Neiguan (PC6), Shenmen (HT7), and Sanyinjiao (SP6) were included based on established clinical practice to synergistically address somatic and autonomic symptoms.

Acupoint locations will follow the GB/T12346–2021 standard (“Naming and Location of Acupoints”). Disposable, sterile stainless steel needles (Tianxie brand, Suzhou Medical Instrument Factory, China; 0.30 mm × 25 mm or 0.30 mm × 40 mm) will be used.

Procedure:

Participants will be treated in a supine position. After routine disinfection of the acupoints with iodine tincture, needles will be inserted to the following depths: 10–20 mm for Sishenzhen, Shenting (GV24), and Yintang (GV29); 25–30 mm for Neiguan (PC6), Shenmen (HT7), and Sanyinjiao (SP6).

Following insertion, gentle lifting, thrusting, and twisting manipulations will be applied to elicit the deqi sensation (defined as soreness, numbness, distension, or a radiating sensation). Once deqi is achieved, all manipulation will cease. The needles will be retained for 30 minutes, during which the participant must remain still.

#### 2.7.2 Waitlist control group

Participants in the waitlist control group will continue their stable, standard medication regimen for the 6-week study period. They will not receive acupuncture or any other study-specific interventions during this time. For ethical reasons, participants in the control group will be offered compensatory acupuncture treatment (using the same protocol as the acupuncture group) after the 6-week intervention period concludes.

To ensure comparability, all participants in both groups must maintain a stable antiparkinsonian medication regimen throughout the study. If any dose adjustments are necessary, they will be assessed by the investigator, documented in the CRF, and converted to the levodopa equivalent dose (LED) (26). No new systemic interventions that could potentially affect depressive symptoms will be permitted during the study. All changes in concomitant medications will be fully documented.

The use and dosage of antidepressant medications will be kept stable whenever possible throughout the 6-week controlled period. “Stable” is defined as no initiation, discontinuation, or dose change within 4 weeks prior to enrollment. During the trial, clinically indicated adjustments will be permitted for safety reasons or symptom worsening. All changes (type, dose, timing, and reason) will be documented and accounted for in the ITT analysis, with sensitivity analyses performed to assess robustness.

To monitor potential contamination during the waitlist period, at each assessment visit participants will be asked using a standardized checklist whether they have received any acupuncture or other non-study interventions for mood or motor symptoms (including acupuncture, electroacupuncture, moxibustion, rTMS, or psychotherapy) since the last visit. Any such use will be recorded as a protocol deviation (type, frequency, timing, and provider) and considered in the per-protocol and sensitivity analyses.

### 2.8 Outcomes

#### 2.8.1 Primary outcome

The primary outcome measure is the change in MADRS total score from baseline (W0) to Week 6 (W6) (27). The MADRS consists of 10 items (total score 0–60) and is highly sensitive to changes in depressive symptoms. Compared to the HAMD, the MADRS places greater emphasis on affective and cognitive dimensions and is less confounded by somatic symptoms (e.g., sleep, appetite, and energy levels) that overlap with PD. This makes it particularly suitable for assessing depressive improvement in patients with PD-D (28).

In addition to analyzing the change as a continuous variable, we will also use the MADRS to calculate the treatment response rate (≥50% reduction) and clinical remission rate (total score ≤10). These outcomes will be interpreted in the context of the minimum clinically important difference (MCID ≈ 2 points) to provide a comprehensive assessment of both statistical and clinical significance (29).

Beyond the primary endpoint at Week 6 (W6), follow-up assessments will be conducted at Weeks 8, 12, and 24 to evaluate the durability and trajectory of treatment effects on depressive symptoms, motor function, sleep quality, and quality of life. These follow-up outcomes are exploratory and will be analyzed descriptively and, where appropriate, using mixed-effects models similar to the primary analysis, without formal adjustment for multiple comparisons.

#### 2.8.2 Key secondary outcomes

To supplement the primary outcome, the key secondary outcome is the change in the Movement Disorder Society Unified Parkinson’s Disease Rating Scale Part III (MDS-UPDRS III) score (30).

The MDS-UPDRS III is an internationally recognized tool for assessing motor function in PD. It consists of 33 items with a total score ranging from 0 to 132, where higher scores indicate more severe motor impairment. In this study, it will be used to evaluate motor function improvement and perform correlation analysis with changes in MADRS scores.

All assessments will be conducted while participants are in their “ON” medication state (60–90 minutes after oral levodopa administration), and the interval since the last dose will be recorded.

#### 2.8.3 Other secondary outcomes

In addition to the core outcomes mentioned above, this study will collect the following secondary outcomes to comprehensively assess the multidimensional effects of the intervention:

Hospital Anxiety and Depression Scale (HADS): This scale consists of two subscales, HADS-Anxiety (HADS-A) and HADS-Depression (HADS-D), with a total of 14 items (7 items per subscale, each subscale scored 0–21). It will be used to supplement the assessment of emotional state, as it is designed to avoid confounding by somatic symptoms (31). 39-item Parkinson’s Disease Questionnaire (PDQ-39): A PD-specific health-related quality of life measure consisting of 39 items across 8 domains. Higher scores indicate poorer quality of life. It will be used to comprehensively evaluate the intervention’s impact on quality of life (32).

Pittsburgh Sleep Quality Index (PSQI): This scale consists of 7 components with a total score ranging from 0 to 21, where higher scores indicate poorer sleep quality. It will be used to evaluate the intervention’s impact on sleep quality over the past month (33).

Patient Global Impression of Change (PGIC): A 7-point Likert scale that will be used for participants to subjectively assess the overall change in their condition, reflecting their perception of treatment effectiveness (34).

Zarit Burden Interview (ZBI): This 22-item scale (total score 0–88) will be used to assess the burden level of primary caregivers. It provides a supplementary measure of the intervention’s impact at the family level (35).

#### 2.8.4 Exploratory mechanistic outcomes

To explore potential biological correlates associated with acupuncture-related changes in depressive symptoms, this study will measure the following serum biomarkers: inflammatory cytokines (IL-6, TNF-α, hs-CRP), brain-derived neurotrophic factor (BDNF), and the kynurenine/tryptophan (KYN/TRP) ratio. Blood samples will be collected at two time points: baseline (W0) and within 24 hours of completing the 6-week treatment (W6). All blood draws will be scheduled between 8:00 and 10:00 AM. Participants will be instructed to fast overnight and to avoid strenuous exercise and alcohol consumption for 24 hours prior to sampling. Among these exploratory biomarkers, brain-derived neurotrophic factor (BDNF) is specified as the primary biomarker of interest, given its established relevance to both depressive symptoms and neuroplasticity in Parkinson’s disease. Inflammatory cytokines (IL-6, TNF-α, hs-CRP) and the kynurenine/tryptophan (KYN/TRP) ratio are considered secondary exploratory biomarkers, intended to provide complementary information on neuroinflammatory and metabolic pathways.

Concentrations of IL-6, TNF-α, hs-CRP, and BDNF will be measured using commercial ELISA kits. The KYN/TRP ratio will be analyzed via liquid chromatography–tandem mass spectrometry (LC-MS/MS). All samples will be processed (centrifuged and aliquoted) within 2 hours of collection, stored at –80°C, and analyzed in a single batch at the end of the study to minimize inter-assay variability. To account for multiple comparisons in exploratory biomarker analyses, false discovery rate (FDR) correction will be applied, and these analyses are intended for hypothesis generation rather than confirmatory inference.

### 2.9 Safety evaluation

All participants receiving at least one intervention will be included in the safety analysis set. Safety monitoring and assessment will be conducted throughout the study. The research team will carefully document all adverse events (AEs), including common acupuncture-related reactions (e.g., local pain, subcutaneous hematoma, or syncope). For each AE, the team will evaluate and track its timing, severity, relationship to the intervention, and final outcome.

In the event of a serious adverse event (SAE)—defined as an event resulting in death, a life-threatening condition, requiring hospitalization, or causing significant disability—the research team will immediately discontinue the intervention, provide necessary medical care, and report the event to the ethics committee within 24 hours. As part of routine safety monitoring, vital signs (temperature, pulse, respiration, and blood pressure) will be recorded at baseline (W0) and Week 6 (W6).

Given the specific risks associated with this study population, the Columbia Suicide Severity Rating Scale (C-SSRS) will be used to systematically assess suicide risk during the screening period and at each follow-up point to ensure participant safety (36).

### 2.10 Statistical analysis methods

The ITT population will include all randomized participants analyzed according to the group to which they were assigned, regardless of adherence. Missing outcome data will be handled under the MMRM framework. A per-protocol (PP) analysis will also be conducted as a sensitivity analysis, excluding major protocol deviations. All statistical analyses will be performed using SPSS Statistics version 26.0 (IBM Corp., Armonk, NY, USA).

Continuous variables will be described as mean ± standard deviation (SD) or median (interquartile range, IQR), while categorical variables will be presented as frequencies and percentages. Baseline characteristics will be compared between groups using appropriate parametric or non-parametric tests.

The primary outcome (change in MADRS score) will be analyzed using a mixed-effects model for repeated measures (MMRM). The model’s fixed effects will include group, time, and the group-by-time interaction. Baseline MADRS score, the stratification factors (baseline severity and antidepressant use), and the Acupuncture Expectancy Scale score will be included as covariates (37). To assess the robustness of the primary findings and to address potential dilution effects associated with very mild depressive symptoms, a prespecified sensitivity analysis will be conducted excluding participants with baseline MADRS scores in the very mild range (e.g., MADRS < 10). The primary analysis model will be re-run in this restricted sample.

Missing values will be assumed to be missing at random (MAR) within the MMRM framework. Sensitivity analyses will be conducted using multiple imputation and pattern-mixture models. Key secondary outcomes (treatment response and remission rates) will be analyzed using chi-square or Fisher’s exact tests. Logistic regression will be used to adjust these outcomes for covariates, and results will be reported as odds ratios (ORs) with 95% confidence intervals (CIs).

Other continuous outcomes (e.g., MDS-UPDRS III, HADS, PDQ-39, PSQI) will be analyzed using ANCOVA or MMRM. Exploratory mechanistic biomarkers will be analyzed using ANCOVA with a false discovery rate (FDR) correction. These exploratory analyses are intended for hypothesis generation only.

Exploratory associations between biomarker changes (W6–W0) and clinical changes will be examined using multivariable regression (and/or partial correlation) models, adjusting at minimum for baseline biomarker level, baseline outcome severity, age, sex, disease duration, antidepressant use, and changes in levodopa equivalent dose (LED), with multiplicity controlled using false discovery rate (FDR), and these analyses are intended for hypothesis generation rather than confirmatory inference.

All tests will be two-sided, with the significance level set at p < 0.05. De-identified participant data will be stored on a secure, password-protected server accessible only to the research team and will be made available upon reasonable request to the corresponding author after publication.

### 2.11 Data collection and management

Research data will be collected using a standardized Case Report Form (CRF) covering demographic information, disease history, scale scores, laboratory results, and adverse events. All scales will be administered by trained investigators in a quiet, private environment. Questionnaires will be collected on-site and immediately checked for completeness.

Data will be double-entered by two research assistants into a password-protected electronic data capture (EDC) system with built-in logic validation. Any inconsistencies will be resolved through third-party verification. The principal investigator will conduct periodic spot checks, and an independent clinical research associate (CRA) will perform scheduled quality audits. All modifications will be recorded in the audit trail.

To protect confidentiality, participants will be identified only by unique codes, and statisticians will remain blinded until the database is locked. Data will be encrypted and stored on restricted servers, while paper CRFs will be kept in locked cabinets for a minimum of 5 years. Following publication, de-identified participant-level data and statistical code will be made available upon reasonable request, in accordance with the Declaration of Helsinki and relevant regulations (38). The full protocol is available upon request. Trial results will be disseminated through peer-reviewed publications and conference presentations.

### 2.12 Quality control and monitoring

All investigators will receive standardized training prior to the trial to ensure protocol adherence, standardized data collection, and consistent reporting of adverse events. Acupuncture procedures will be performed exclusively by licensed acupuncturists following strict standard operating procedures (SOPs). Adherence will be documented through attendance logs. Good adherence is defined as the completion of ≥80% of scheduled treatment sessions. All protocol deviations or discontinuations will be recorded and addressed in the final analysis.

An independent clinical research associate (CRA), operating independently of the research team, will periodically review study progress, safety, and data quality. Core outcome measures (MADRS, MDS-UPDRS III) will be assessed by trained, blinded evaluators. Efforts will be made to standardize assessment time points and, where possible, maintain the same evaluator for each participant’s follow-up visits to minimize bias.

As a single-center study, daily management is coordinated directly by the principal investigator without a separate steering committee. Given the low-risk nature of acupuncture, a formal Data Monitoring Committee (DMC) is not established, and no interim analysis is planned.

## 3 Discussion

This study aims to evaluate the efficacy and safety of acupuncture as an adjunctive therapy for patients with depression in PD-D. A key innovation of this trial is its systematic integration of multidimensional clinical outcomes with a panel of serum biomarkers reflecting shared pathological mechanisms: inflammatory cytokines, brain-derived neurotrophic factor (BDNF), and the kynurenine/tryptophan (KYN/TRP) ratio. This design is intended to facilitate a more integrated evaluation of acupuncture’s clinical effects and their potential biological correlates in PD-D, thereby enhancing the understanding of the potential neuro-immune-endocrine mechanisms underlying concurrent improvements in mood and motor function.

Depression in PD is not only highly prevalent but also strongly associated with motor impairment (39). Extensive neuroimaging and neurobiological studies indicate that the two conditions share highly convergent pathological mechanisms, including serotonin and norepinephrine system dysfunction, chronic neuroinflammation, hypothalamic-pituitary-adrenal (HPA) axis hyperactivation, and decreased brain-derived neurotrophic factor (BDNF) levels. This evidence suggests that interventions targeting only affective or motor symptoms in isolation are unlikely to break the maladaptive bidirectional relationship between them. Therefore, there is an urgent need for comprehensive intervention strategies designed to holistically modulate these shared pathological pathways.

Acupuncture, a safe and well-tolerated non-pharmacological therapy, has gained increasing attention in the comprehensive management of PD in recent years. Previous clinical studies have suggested that acupuncture can improve multiple non-motor symptoms in PD. Consistently, our earlier clinical studies also demonstrated that acupuncture effectively alleviates anxiety, improves sleep and gastrointestinal symptoms, and enhances UPDRS scores, thereby contributing to better overall quality of life in patients with PD (40–42).

Furthermore, electroacupuncture has been reported to alleviate depressive symptoms in patients with Parkinson’s disease with depression (PD-D) and to increase serum brain-derived neurotrophic factor (BDNF) levels, demonstrating superior therapeutic effects compared with pharmacotherapy alone (43). These findings imply that acupuncture may not only target mood symptoms but also exert indirect benefits on motor function. However, most existing evidence comes from small-sample, exploratory trials with methodological limitations. High-quality studies that integrate clinical outcomes with mechanistic biomarkers remain scarce, underscoring the need for further validation (18, 44).

Previous randomized controlled trial protocols have proposed the use of sham acupuncture procedures—such as shallow insertion, absence of deqi, or no needle manipulation—as methodological options to reduce nonspecific effects within assessor-blinded designs (45). In contrast, the present trial adopts a waitlist control to evaluate the short-term adjunctive effectiveness of acupuncture in a real-world clinical context. Accordingly, this study aims to assess the clinical effectiveness of adjunctive acupuncture in addition to stable standard pharmacotherapy.

Therefore, a waitlist control design was adopted. This approach ensures ethical compliance, enhances patient acceptability, and more closely reflects real-world clinical practice, thereby improving both feasibility and interpretability. Given the inherent challenges in implementing double-blind procedures for both practitioners and participants in acupuncture trials, this design is considered practical, appropriate, and scientifically justified.

Second, stratified randomization will be used to control for key prognostic factors, and blinding will be maintained during both outcome assessment and data analysis to minimize the risk of bias. Furthermore, the outcome measures balance scientific rigor with clinical relevance. The MADRS was selected as the primary endpoint because it avoids confounding by PD-related somatic symptoms. This primary outcome, along with the Hospital Anxiety and Depression Scale (HADS), Pittsburgh Sleep Quality Index (PSQI), MDS-UPDRS III, and quality of life scales, forms a comprehensive evaluation system. This system covers mood, motor function, sleep, and quality of life, allowing for a more complete reflection of acupuncture’s clinical value.

For our mechanistic exploration, we selected three categories of serum biomarkers: inflammatory cytokines (IL-6, TNF-α, hs-CRP), BDNF, and the KYN/TRP ratio. These were chosen to map onto neuroinflammation, neuroplasticity, and the tryptophan metabolic pathway, respectively. Elevated inflammatory factors can exacerbate emotional and motor impairments by disrupting monoamine function and accelerating neuronal degeneration (46). Reduced BDNF limits emotional regulation and motor learning capacity (47).

Furthermore, a KYN/TRP imbalance not only reduces serotonin synthesis substrates but also promotes the accumulation of neurotoxic metabolites, such as quinolinic acid. This process bidirectionally drives both depressive and motor impairments (48). Therefore, these biomarkers serve as pivotal links between depression and motor impairment and may also function as objective measures of acupuncture’s effects. If acupuncture can simultaneously alleviate clinical symptoms and modulate these biological pathways, this would provide preliminary, hypothesis-generating evidence that acupuncture may attenuate the bidirectional interplay between emotional and motor symptoms (49).

The strengths of this study include its randomized controlled design, stratified randomization based on baseline depression severity and antidepressant use, assessor blinding, and the integration of multidimensional clinical outcomes with mechanistic biomarkers reflecting neuroinflammation, neuroplasticity, and tryptophan metabolism. These features allow for a comprehensive evaluation of the clinical effectiveness of acupuncture and its potential biological correlates in Parkinson’s disease with depression.

Several limitations should also be acknowledged. First, as a single-center trial with a modest sample size, the generalizability of the findings may be limited. Second, the use of a waitlist rather than a sham acupuncture control, while ethically and pragmatically justified for evaluating add-on effectiveness in a real-world setting, precludes blinding of participants and acupuncturists and therefore limits the ability to fully disentangle acupuncture-specific effects from non-specific factors (e.g., placebo responses, contextual influences, and expectancy effects), particularly for affective outcomes. We acknowledge that the inclusion of participants with very mild depressive symptoms may attenuate observed effect sizes. However, depressive symptoms in Parkinson’s disease are often mild but persistent, clinically meaningful, and associated with functional impairment. The planned sensitivity analyses excluding very mild cases are intended to evaluate the robustness of treatment effects across different baseline severity levels.

Although baseline expectations will be assessed using the Acupuncture Expectancy Scale and included as covariates in the primary and sensitivity analyses, this approach can only partially address expectancy-related bias rather than eliminate it. Accordingly, the results will be interpreted primarily as evidence of adjunctive effectiveness of acupuncture in addition to standard pharmacotherapy, rather than acupuncture-specific efficacy in isolation.

In summary, this study protocol builds upon previous research by improving its design, outcome measures, and mechanistic exploration. By integrating clinical outcomes with mechanistic biomarkers, the study aims to systematically evaluate the adjunctive effectiveness and biological correlates of acupuncture in managing PD-D.

If acupuncture can simultaneously alleviate clinical symptoms and modulate these biological pathways, this would support the hypothesis that it may contribute to disrupting the maladaptive bidirectional relationship between emotional and motor symptoms.
